# Case Report: Prepubertal-type testicular teratoma with local metastasis in a postpubertal patient

**DOI:** 10.3389/fonc.2025.1547258

**Published:** 2025-02-25

**Authors:** Olivia C. Ghirardelli Smith, Alexander K. Tsai, Minghao Zhong, Pegah Dejban, Andrew C. Nelson, Michelle Dolan, Emmanuel S. Antonarakis, Paari Murugan

**Affiliations:** ^1^ Department of Medicine, Division of Hematology, Oncology & Transplantation, University of Minnesota, Minneapolis, MN, United States; ^2^ Department of Laboratory Medicine and Pathology, University of Minnesota, Minneapolis, MN, United States; ^3^ Department of Pathology and Laboratory Medicine, Henry Ford Health, Detroit, MI, United States

**Keywords:** germ cell neoplasia *in situ* (GCNIS), germ cell tumor (GCT), prepubertal-type teratoma, postpubertal-type teratoma, isochromosome 12p (i12p), testicular cancer

## Abstract

**Introduction:**

We report for the first time a case of a postpubertal patient presenting with a metastatic prepubertal-type testicular teratoma.

**Case discussion:**

A 29-year-old male with a history of corrected unilateral cryptorchidism presented with progressive bilateral lower extremity edema. Imaging revealed an inferior vena cava thrombus associated with a complex mass. A left testicular ultrasound identified a solid lesion suggestive of a germ cell tumor, leading to a left radical orchiectomy, which revealed a mature pure teratoma with no evidence of germ cell neoplasia *in situ* (GCNIS). Excision of the retroperitoneal mass confirmed the presence of mature teratomatous elements without evidence of non-teratomatous germ cell tumor elements or cytological atypia. Fluorescence *in situ* hybridization (FISH) showed no evidence of gain of 12p, and next-generation sequencing showed no alterations in genes known to be associated with GCT.

**Conclusion:**

This case illustrates that pure mature teratomas lacking chromosome 12p abnormalities, GCNIS, and other dysgenetic features, occurring in postpubertal males, cannot invariably be classified into the benign prepubertal-type teratoma category. Contrary to current paradigm, in rare cases these may represent tumors with metastatic potential.

## Introduction

Testicular tumors are primarily germ cell in origin. Germ cell tumors (GCT) are classified into germ cell neoplasia *in situ* (GCNIS)-derived and GCNIS-unrelated tumors. GCNIS-derived tumors include seminomatous, non-seminomatous (NSGCT) and mixed germ cell tumors. Non-GCNIS-derived tumors include prepubertal-type teratomas, spermatocytic tumors and yolk sac tumors. NSGCTs form choriocarcinomas, embryonal carcinomas, yolk sac tumors, or teratomas, often presenting as “mixed” tumors with multiple histologic subtypes. A minority are “pure,” composed of a single histology. Recent WHO classification updates for pure testicular tumors in adults distinguish between the rarer prepubertal-type teratomas (Type 1 TT) from the more common and aggressive postpubertal-type teratomas (Type 2 TT) (1). Type 1 TT are considered benign and are therefore thought to have no metastatic potential, however their clinical behavior in postpubertal men remains elusive owing to their rarity. We report for the first time a case of a postpubertal patient presenting with a metastatic prepubertal-type testicular teratoma.

## Case description

**Figure 1:**
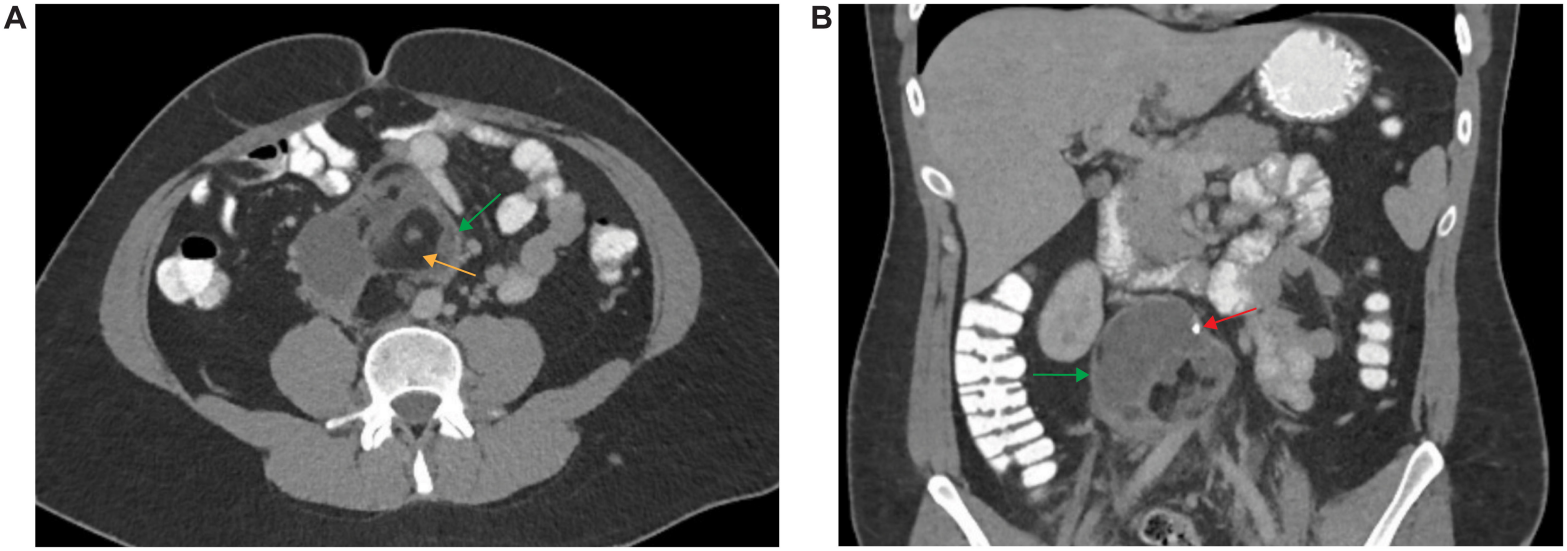
Imaging of Retroperitoneal Mass. CT angiogram of the chest, abdomen, and pelvis with axial **(A)** and coronal **(B)** sections depicting a retroperitoneal mass (yellow arrows) containing macroscopic fat (orange arrow) and coarse calcifications (red arrow).

A 29-year-old male with a past medical history of unilateral cryptorchidism corrected via orchidopexy at age 2, presented with subacute, progressive bilateral lower extremity edema. CT angiogram of the chest, abdomen, and pelvis revealed a thrombus involving the inferior vena cava (IVC). A complex mass-like structure containing macroscopic fat and coarse calcifications measuring 9 x 9 x 8.4 cm appeared to be associated with the IVC thrombus and displacing the aorta (**Figure 1**). The mass was thought to represent IVC rupture with associated hematoma versus a neoplasm. Testicular ultrasound revealed a normal right testicle and a left testicle containing a lobulated and partially calcified solid lesion measuring 1.3 cm, suggestive of a germ cell tumor. Alpha-fetoprotein (AFP) and beta-human chorionic gonadotropin (hCG) levels were normal and lactate dehydrogenase (LDH) was slightly elevated (331 U/L, reference range 85-227).

**Figure 2:**
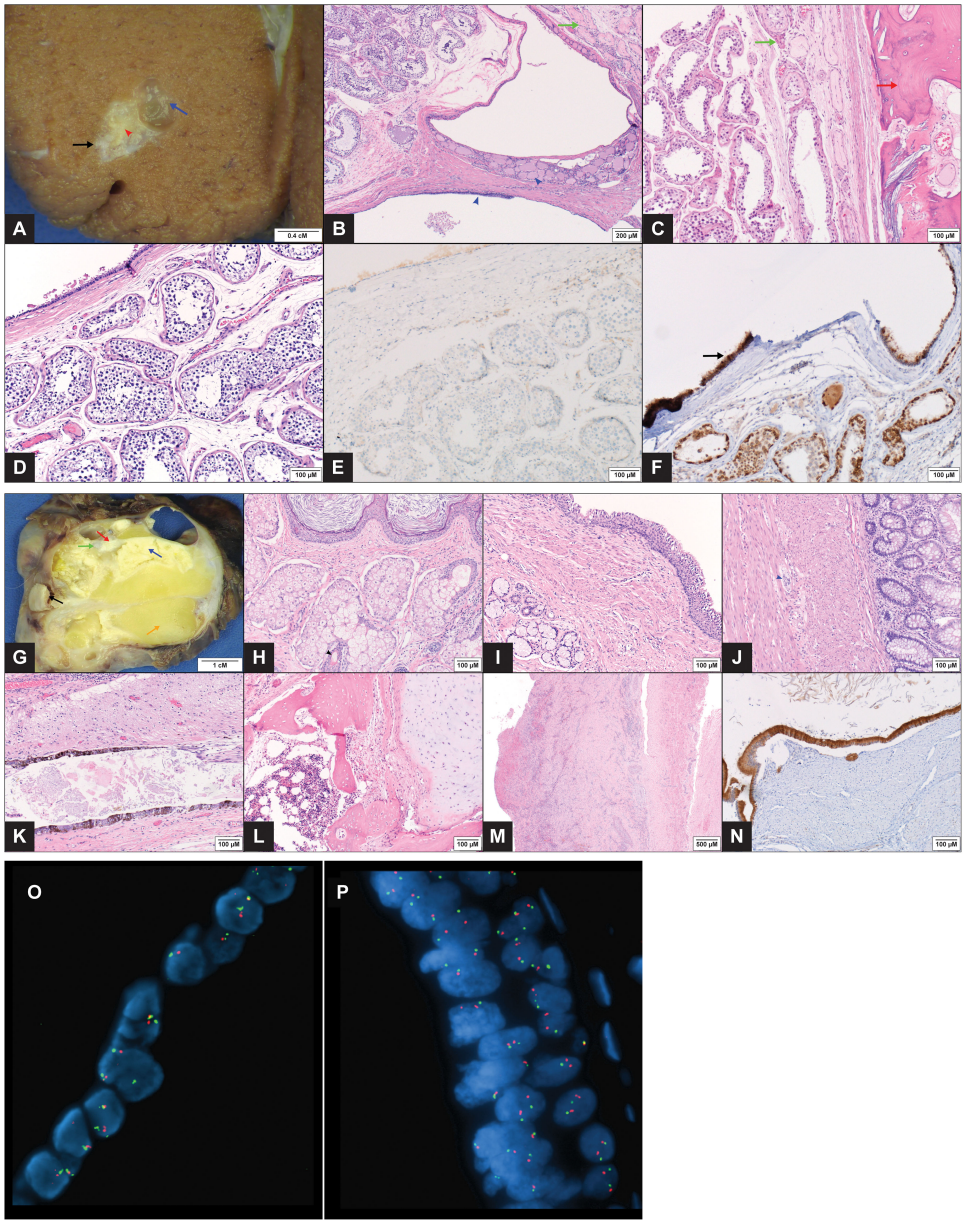
Pathology Images of Testicular and Retroperitoneal Tumors **(A–F)** Gross image of the testicular tumor (1 x 0.9 x 0.5 cm) with a mucus containing simple cyst (blue arrow) and a tan, firm focus representing bone (red arrow head). A zone of perilesional fibrosis immediately adjacent to the mass is seen (black arrow). The adjacent testicular parenchyma appears grossly normal **(A)**. Microscopic exam showed mature teratoma elements consisting of a respiratory epithelium lined simple cyst with few thyroid follicles (blue arrow head) (**B**, H&EX100) and lamellar bone (red arrow) (**C**, H&EX200). Fibrosis and atrophic seminiferous tubules are present in areas immediately adjacent to the tumor (green arrows) **(B, C)**. Teratomatous ciliated respiratory epithelium (top left) alongside normal seminiferous tubules with complete spermatogenesis, devoid of ‘dysgenetic’ features (**D**, H&EX200). Immunohistochemical stains showing negative OCT4 IHC in the seminiferous tubules (**E**, IHCX200) while IMP3 demonstrates strong reactivity in the teratoma epithelium (arrow) (**F**, IHCX200). **(G–N)** Gross image of the retroperitoneal mass (11.2 x 8.4 x 7.3 cm) demonstrating multiple structures, including dermoid cyst with hair (black arrow), keratinous material (blue arrow), cartilage (green arrow), bone (red arrow), adipose tissue (orange arrow) and multiple cysts **(G)**. Microscopic images showing dermoid cyst components with keratinizing squamous epithelium (top), and sebaceous glands (bottom) with hair follicle (black arrow head) (**H**, H&EX200). Other organoid structures included bronchial wall elements with ciliated epithelium (top right) and underlying smooth muscle layer and mucous glands (**I**, H&EX200); colonic wall elements with goblet cell glands (right) and underlying dual smooth muscle layers with ganglion cells in between (blue arrow head) (**J**, H&EX200). Multiple other mature tissue types, including glial (top), pigmented neuroepithelium (bottom) (**K**, H&EX200), cartilage (right) (**L**, H&EX200) and bone with marrow tissue (left) (**M**, H&EX200) were also present. The attached IVC (right) showed an organizing blood clot (left) with reactive changes, adventitial adhesions and no evidence of malignancy (**M**, H&EX40). Immunohistochemical stain for IMP3 with diffuse strong reactivity in the teratomatous epithelium (**N**, IHCX200). **(O, P)** FISH performed with probes (Abbott Molecular, Abbott Park, IL) to the 5’ (telomeric) portion of ETV6 (12p13.2) (red) and the centromere of chromosome 12 (12p11.1-q11) (green). FISH performed on formalin-fixed, paraffin-embedded tissue from the testicular tumor **(O)** and the retroperitoneal mass **(P)** showed the lesional cells to have an equal number of red and green signals, consistent with the absence of an isochromosome 12p. In addition, there is no evidence of 12p gain.

A left radical orchiectomy was performed the following day. Given concern for metastatic GCT, the patient also received a single dose of bleomycin, etoposide, and cisplatin (BEP) chemotherapy. Histopathology revealed a 1 x 0.9 x 0.5 cm mature teratoma composed of a respiratory epithelium-lined cyst with adjoining bone and thyroid follicles (**Figures 2A–D**). There was no evidence of non-teratomatous GCT elements, cytological atypia, microcalcifications, GCNIS, lymphovascular invasion or extratesticular extension. A focal area of parenchymal scarring with few atrophic seminiferous tubules was identified immediately adjacent to the tumor. Additionally, the seminiferous tubules revealed normal, active spermatogenesis and normal basement membranes, without dysgenetic features. Immunohistochemical staining for OCT4 was negative for GCNIS, no SALL4 reactivity was present in the tumor, and IMP3 immunohistochemical stain was strongly positive in the epithelial elements (**Figures 2E, F**). In light of the clinicopathologic findings of a pure teratoma without evidence of secondary somatic-type malignancy, chemotherapy was discontinued and retroperitoneal lymph node dissection (RPLND) was performed. A 11.2 x 8.4 x 7.3 cm retroperitoneal mass extending from the infrarenal portion of the aorta to the common iliac arteries was excised. The mass was adherent to the aortic wall 3 cm below the renal arteries. There was also an associated complete occlusion of the infrarenal IVC to the level of the common iliac vein. Histopathologic analysis of the retroperitoneal mass showed only mature teratomatous elements including dermoid cyst components with hair, organoid respiratory tissue, organoid gastrointestinal tissue, glial, neuroepithelial and choroid plexus tissue, pancreatic tissue, fibroadipose tissue, smooth muscle, cartilage, and mature bone with hematopoietic elements (**Figures 2G–M**). The vascular lesion showed features of an organizing thrombus with no evidence of GCT elements. Chemotherapy related treatment effect was not identified. IMP3 immunohistochemical stain demonstrated strong reactivity in the epithelial elements (**Figures 2N**).

FISH analysis was performed on both masses to evaluate for gain of chromosome 12p, as would result from the presence of an isochromosome 12p (i(12p)). 100-200 interphase cells were analyzed using probes to 5’ (telomeric) ETV6 (12p13.2) and D12Z3 (12p11.1-q11). No evidence was found in either specimen of gain of 12p relative to the chromosome 12 centromere (**Figure 2O, P**). An additional FISH assay performed on the retroperitoneal mass using PLP2 (12p11.2) and D12Z3 probes was also negative for gain of 12p.

Whole exome next generation sequencing (WES-NGS) (Caris Life Sciences, Phoenix, AZ) was performed on the retroperitoneal mass (the limited size of the testicular specimen was insufficient for analysis) and showed no evidence of detectable pathogenic or likely pathogenic alterations; specifically, there was no evidence of alterations in genes in 12p nor in genes known to be associated with GCT. The tumor mutational burden (TMB) was 4 mutations/Mb and the tumor was microsatellite stable (MSS) by NGS. Genome-wide loss of heterozygosity (gLOH) was low at 2%. No gene-level amplifications or deletions were detected by NGS. Inference of genome wide copy number (CN) alterations from WES-NGS suggested low level gains of 3q, 5q, 6p/6q, and portions of 7p/7q coupled with single copy deletion of 18p/18q and 20p/20q. Heterogeneous CN estimation across chromosome 12 did not modify the conclusion of unaltered 12p copy number and lack of i12p reported by the FISH studies.

The patient received no additional tumor-directed therapies and surveillance imaging remains negative for recurrent disease to date, over five years since diagnosis at the time of publication.

## Discussion

We report a postpubertal patient with a testicular tumor meeting the morphological and cytogenetic criteria of a mature pure Type 1 TT, presenting with metastasis to the retroperitoneum. To our knowledge this is the first such reported case. Although there have been multiple documented cases of metastatic mature pure testicular teratoma in adults, none have been reported to fit the definition of a Type 1 TT, i.e. absence of chromosome 12p abnormalities, GCNIS and testicular dysgenetic features (2–4). Despite two documented examples of metastatic pure Type 1 TT in the pediatric age group, both cases were diagnosed as immature teratomas (5, 6).

**Figure 3:**
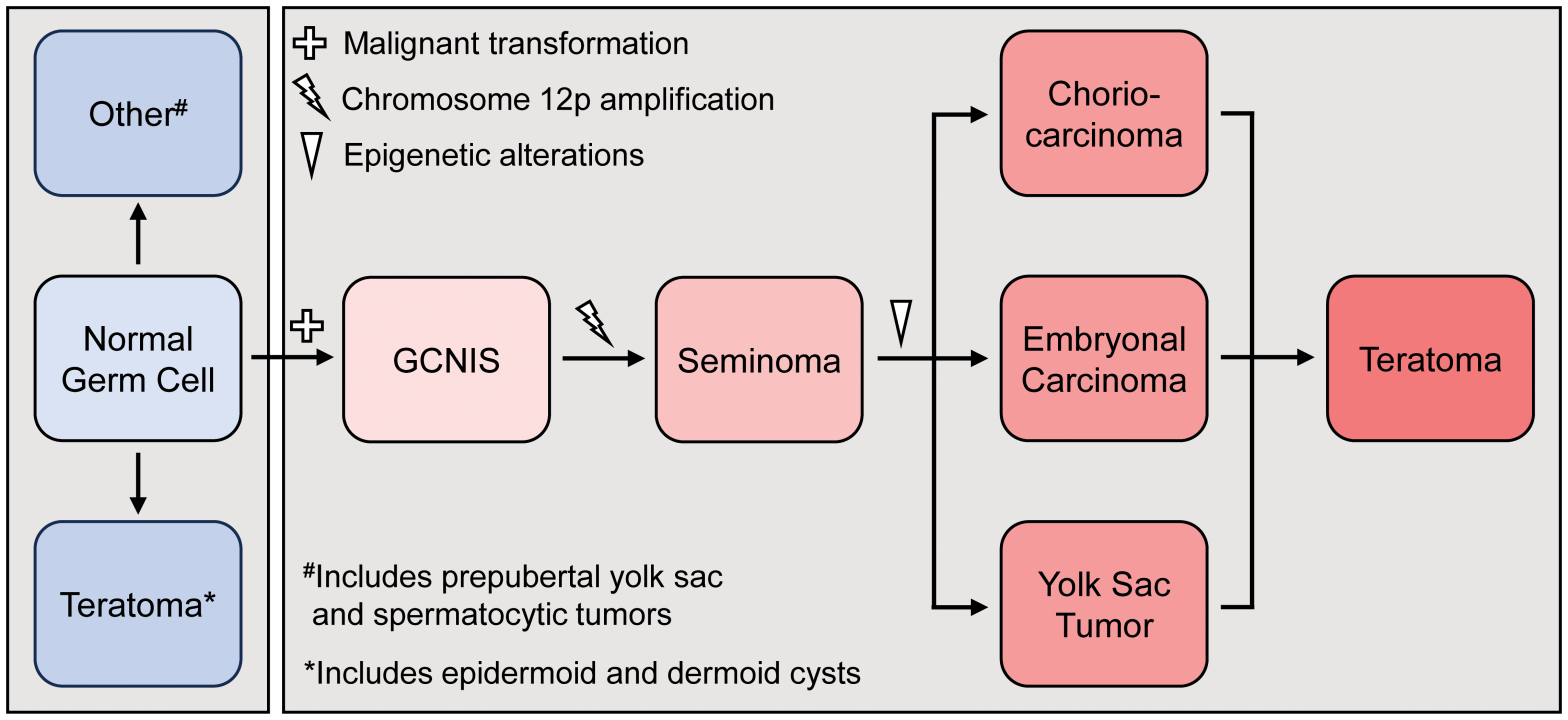
Histogenetic model for the development of pre-pubertal (blue) and post-pubertal (red) teratoma development. GCNS=germ cell neoplasia *in situ*.

Historically, teratomas found in the postpubertal testes have been considered malignant, supporting the histogenetic model of teratoma development (**Figure 3**) (7–9). Persistently immature germ cells arrested at the gonocyte stage undergo malignant transformation due to aberrant activation of the KITLG/KIT pathway and overexpression of transcription factors such as NANOG and OCT4 (10, 11). According to this histogenetic model, pubertal activation of the hypothalamic-pituitary-gonadal axis leads to proliferation of these previously dormant transformed gonocytes, giving rise to GCNIS and differentiation into seminomas due to gain of chromosome 12p. Seminomas can be further reprogrammed into multiple histological GCT types, including postpubertal-type teratomas, through epigenetic regulation, particularly via DNA methylation (1, 8, 12) (**Figure 3**). In contrast, Type 1 TT are postulated to develop from gonocytes that undergo reprogramming, resulting in reversal of germline specification, without malignant transformation. They are typically diploid, not associated with GCNIS, lack chromosome 12p abnormalities and do not harbor driver mutations (13–15). Several case series and reports detailing approximately 50 cases in the last two decades identified teratomas in postpubertal men that did not meet malignant criteria and instead appeared to reflect benign, Type 1 TT (16), leading to a reevaluation of the WHO testicular cancer classification system in 2016 (13, 17). It has since been proposed that Type 1 TT found in postpubertal men represent late-appearing benign pediatric tumors that have arisen from reprogrammed non-malignant germ cells (1, 7). However, the substantial incidence of these tumors in patients beyond the second or third decade of life suggests the process may also occur *de novo* during adult life (2, 13, 17). Unlike Type 1 TT, postpubertal-type teratomas, even in the pure form are associated with a high risk of recurrence and metastasis (20-46% cases) (4, 18).

In the updated WHO classification system, Type 2 TT fall within the GCNIS-derived tumor category, whereas Type 1 TT fall within the non-GCNIS-derived category (1). Multiple morphological and molecular features aid in distinguishing Type 1 TT from Type 2 TT in postpubertal men (**Table 1**).

**Table 1:** Molecular and morphological features of post- and pre-pubertal type teratomas.

	Postpubertal type	Prepubertal type
GCNIS	90% of cases (8)	–
Tubular atrophy/scarring	Frequent (8)	–
Microlithiasis	+/-	–
Impaired spermatogenesis	+/-	–
Cytological atypia	Frequent (8)	–
12p overexpression	80-90% of cases (2)	–

Sources: (1, 2, 8), and (13).

The presence of GCNIS and testicular dysgenetic features, including tubular atrophy, peritubular sclerosis, Sertoli cell-only tubules, microlithiasis, and impaired spermatogenesis are morphological exclusion criteria for the diagnosis Type 1 TT (1). In contrast, Type 2 TT often display dysgenetic testicular features as well as evidence of either GCNIS and/or GCT regression (also known as a “burned out” tumor) including zones of scarring, intratubular coarse calcifications, necrosis, lymphoplasmacytic infiltrates and hemosiderin deposition (1, 7).

Molecularly, the presence of chromosome 12p overexpression, either by gene amplification or resulting from the presence of an isochromosome 12p, is considered pathognomic for a postpubertal-type GCNIS derived malignant germ cell tumor, including teratomas. Several genes in 12p, including NANOG, CCND2, KRAS, KITLG and BCAT1, are postulated to contribute to tumorigenesis. Abnormalities in multiple other genes and distinct patterns of global DNA methylation have also been described. In addition, these tumors are typically aneuploid and commonly harbor recurrent chromosomal deletions and amplifications (12, 19, 20). In contrast, Type 1 TT rarely contain chromosome 12p abnormalities, are typically diploid, do not harbor driver mutations and demonstrate normal DNA methylation patterns (2, 14, 15, 21, 22). FISH is the gold standard for detection of copy number gain of chromosome 12p (19, 23). IMP3, a member of the insulin-like growth factor II mRNA-binding family is an oncofetal protein that has an important role in embryogenesis and carcinogenesis. IMP3 overexpression has been identified in several malignancies, including nearly 100% of primary and metastatic postpubertal-type testicular mature teratomas. In contrast, all benign ovarian mature teratomas and prepubertal-type benign testicular teratomas developing in patients older than 14 months were negative for the marker (3, 21, 24).

Despite being considered a benign neoplasm, evidence in this case points to the development of a heretofore unreported metastatic Type 1 TT, with several features strongly supporting a Type 1 TT over a malignant Type 2 TT. Morphologically, there was no evidence of GCNIS (confirmed by the absence of OCT4 IHC). The seminiferous tubules were normal-appearing and exhibited active spermatogenesis without basement membrane thickening, tubular atrophy, microcalcifications or other features of testicular dysgenesis. Apart from a small area of perilesional mass-related fibrosis, no scarred areas suggesting burned out GCT or chemotherapy related changes were identified. No immature elements or cytologic atypia were present in either the testicular or retroperitoneal tumors. Multiple morphologic features that are encountered more commonly in Type 1 TT compared to Type 2 TT, such as organoid structures (including dermoid components), mature bone formation and preponderance of respiratory over gastrointestinal type epithelium were present in these tumors (3). Moreover, grossly identifiable hair was present in the retroperitoneal tumor, a finding never reported in Type 2 TT. Molecularly, the lack of 12p amplification [including rare amplication restricted to chromosomal bands 12p11.2-p12.1 (25)], the absence of identifiable pathogenic genetic alterations by comprehensive NGS and a very low gLOH score support a Type 1 TT, given that Type 2 TT frequently harbor large-scale chromosomal losses and gains (most commonly amplifications in 12p) with elevated gLOH (26–29). Finally, the patient’s clinical course would be atypical for Type 2 TT, given lack of recurrent disease over five years since his diagnosis, with only a single dose of BEP chemotherapy followed by surgical resection of the primary and retroperitoneal tumors. The premise that this Type 1 TT exhibited metastatic/malignant behavior is supported by the presence of a synchronous retoperitoneal tumor demonstrating similar histological features in the landing site of testicular primaries. In addition, the presence of diffuse IMP3 protein expression in the retroperitoneal and testicular tumors of this postpubertal patient suggests malignancy in both. Furthermore, despite the low TMB and gLOH scores and the lack of an i(12p), NGS detected multiple whole chromosome and copy number gains and losses in the retroperitoneal tumor, consistent with a complex karyotype that is rarely seen in benign Type 1 TT.

Nevertheless, given the highly unusual nature of this case, discussing the unlikely possibility of synchronous benign primaries is warranted. Although contemporary thought is that retroperitoneal GCTs in the postpubertal male are virtually always metastatic from a testicular primary, there are two rare possibilities that may account for the presence of a primary prepubertal-type GCT in the retroperitoneum: 1) An extragonadal retroperitoneal prepubertal-type GCT arising from mismigrated primordial germ cells to the midline retroperitoneum during embryogenesis (30). These reprogrammed tumor cells are primed for differentiation into somatic lineages and commonly result in the formation of mature teratoma with increasing age. 2) A gonadal retroperitoneal prepubertal-type GCT arising from a supernumerary testis in the abdominal cavity. Polyorchidism likely results from transverse division of the urogenital ridge during 4-6 weeks of gestation and can occur in association with cryptorchidism, as was present in this patient (31).

The relative risk of testicular malignancy in males with history of cryptorchidism is approximately 3-8 times that of those with normal testicular development (32). The patient’s history of cryptorchidism likely contributed to teratoma development, although it is typically associated with Type 2 TT.

In conclusion, we report for the first time a case of a postpubertal patient presenting with a metastatic prepubertal-type testicular teratoma. Although likely rare, this case suggests tumors that satisfy the current criteria for benign prepubertal-type teratoma may show malignant/metastatic progession. The presence of copy number abnormalities without 12p gain and other known genomic alterations suggests these tumors develop via an as yet unknown alternative mechanism. Nevertheless, the indolent clinical behavior appears comparable to entities such as benign metastasizing leiomyoma that consistently harbor genomic abnormalities distinct from fully malignant leiomyosarcoma (33). The case also stresses the importance of multidisciplinary decision-making and emphasizes the significance of awaiting pathological findings prior to deploying cancer-directed treatments.

## Data Availability

The original contributions presented in the study are included in the article/supplementary material. Further inquiries can be directed to the corresponding author/s.
